# Orthodontic diagnosis and usability of the web-based e-learning application orthotrainer

**DOI:** 10.1186/s12909-025-07997-9

**Published:** 2025-10-02

**Authors:** Norbert Alexander Lang, Franziska Alina Lang, Martin Lemos, Laura Bell, Daniel Fink, Christian Niederau, Katharina Mücke, Kathrin Becker, Teresa Kruse, Bert Braumann, Michael Wolf, Isabel Knaup

**Affiliations:** 1 Department of Orthodontics and Dentofacial Orthopedics, Medical Faculty, RWTH Aachen University, Aachen, Germany; 2 Audiovisual Media Center, Medical Faculty, RWTH Aachen University, Aachen, Germany; 3 Department of Orthodontics and Dentofacial Orthopedics, Medical Faculty, Heinrich Heine University, Düsseldorf, Germany; 4 Department of Orthodontics and Dentofacial Orthopedics, Medical Faculty, Charité, Berlin, Germany; 5 Department of Orthodontics and Dentofacial Orthopedics, Medical Faculty Cologne University, Cologne, Germany

**Keywords:** Technology-enhanced learning, Digital dental education, E-learning, Hybrid learning, Teaching innovation, Dental curriculum

## Abstract

**Background:**

In orthodontic education, the integration of digital teaching methods is crucial to meet the complex demands of the discipline. This study evaluates the performance of the web-based e-learning application Orthotrainer, designed for orthodontic case planning. It is hypothesised that case evaluations through this digital approach are comparable to traditional analogue measurement methods. The aim is to contribute to the standardisation of orthodontic learning content in North Rhine-Westphalia and promote innovative teaching strategies.

**Methods:**

A total of 61 dental students were randomly assigned to either an analogue (group A) or a digital (group D) cohort. The students were tasked with performing orthodontic case planning, which included model analysis and X-ray analysis, specifically OPG and LCR analysis. As part of a crossover design, both groups completed both a digital and an analogue treatment case. The quality and usability of the digital application were evaluated, and the results of the learning modes were quantitatively compared.

**Results:**

In the model analyses the tooth-width measurements were almost identical between analogue and digital methods, with only minimal differences observed. Analogue techniques demonstrated slightly higher accuracy in arch-width determinations, while the digital approach exhibited superiority over analogue measurements in classifying canine Angle relationships. Conversely, analogue gauge measurements maintained their superiority in molar classification In the radiographic assessment, the Orthotrainer demonstrated its superiority, with its lateral cephalometric radiograph landmarking (LCR) success rate exhibiting a significant increase in comparison to conventional methods (LCR: 55.74% vs. 46.80%). Both groups completed the case planning in the allotted time, with no significant differences. Finally, students provided a favourable evaluation of the Orthotrainer, with a mean System Usability Scale score of 77.62 (SD 23.16, 95% confidence interval [CI], 71.70–83.56).

**Conclusions:**

The Orthotrainer provides diagnostic performance that is equivalent to that of traditional analogue case assessment, particularly regarding cephalometric analysis. It was also positively received by students in terms of diagnostic performance and user-friendliness, which indicates its potential as a reliable supplement to conventional teaching methods.

**Supplementary Information:**

The online version contains supplementary material available at 10.1186/s12909-025-07997-9.

## Background

In orthodontic education, leveraging swiftly evolving technologies is essential because of the inherent complexities of teaching this discipline. Crafting an effective orthodontic treatment plan requires careful analysis of diverse diagnostic information, including patient histories, radiographic images, and study models [1]. Educating students in orthodontics poses unique challenges, as both qualitative clinical evaluations and quantitative assessments of intricate craniofacial structures are needed. Diagnostic tools, such as orthopantomogram (OPG) evaluation, cephalometric analyses, and three-dimensional model analyses, are essential for comprehensive treatment planning. Despite the clear potential of digital teaching methods to support these demanding processes, such approaches have thus far been underutilised in orthodontic training [2]. Considering the global challenges in dental education—further exacerbated by the COVID-19 pandemic—innovative e-learning strategies and hybrid teaching models offer a significant opportunity to equip students with the advanced competencies required for modern orthodontics [3–5]. Numerous studies and systematic reviews have proven the effectiveness of digital teaching methods such as e-learning, apps, AI-supported tools and digital models. Particularly when teaching theoretical content and practical skills—for example, in lateral cephalometric radiographs (LCRs)—these approaches have been shown to be at least equivalent, and in some cases even superior, to traditional forms of teaching [6–9]. Previous online tools for orthodontics – such as an online cephalometry trainer [10] and virtual training courses for surgical planning [11] – have demonstrated their potential and influence on learning methods [12]. Orthotrainer expands on these approaches by combining the analysis of intraoral 3D models, OPG evaluation and LCR landmarking in a single web application, thus providing a more comprehensive environment for case planning. In addition to orthodontics, digital teaching aids and virtual reality simulators have also been successfully used in training in local anaesthesia [13], endodontics [14], paediatric dentistry [15] and oral radiology [16], demonstrating their broad potential for improving the acquisition of clinical skills in all dental courses. These technology-assisted learning approaches are particularly valuable for procedures such as the administration of local anaesthesia or initial endodontic access, where direct practical training on living patients is not possible. Investigating the influence of learning modalities on a dental student’s ability to assimilate new information and apply it in clinical contexts could demonstrate the potential benefits of dedicating faculty resources to the creation of clinically relevant e-learning activities [16–18]. Acceptance among students is also high: many favour digital learning methods or at least see them as useful additions to face-to-face teaching [9, 19]. Digital methods are generally not seen as a complete replacement but rather as a supporting element in the overall didactic approach. The spread of digital models and tools is steadily increasing, even if they are not yet established across the board. In North America, for example, approximately 35% of postgraduate programmes predominantly use digital models, and the trend continues to increase [2, 9].

However, full penetration and routine use in all training centers has not yet been achieved, meaning that the statement of underutilisation to date is true in part, but the trend is clearly moving in the direction of increased integration of digital teaching methods [9, 12]. The objective of this study is to evaluate the effectiveness of e-learning in orthodontic education by analysing the applicability and practicality of the web-based e-learning application Orthotrainer, a novel application for orthodontic case planning designed for educational purposes. This study addresses unmet needs in orthodontic education by leveraging integrated OPG and 3D model analyses to standardise diagnostic workflows, support self-directed learning, and improve student satisfaction with case-planning exercises. It is hypothesised that an evaluation of a case via the e-learning approach is comparable to a conventional evaluation via analogue measurement methods and that, as part of the multicentric, freely available OrthoCampus platform, Orthotrainer will contribute to the standardisation of orthodontic learning content in North Rhine-Westphalia in the future.

## Method

This randomised prospective crossover study received approval from the Ethics Committee at RWTH Aachen University Hospital (EK 24/209). The study was conducted between April 2023 and July 2024 in full compliance with the ethical standards outlined in the World Medical Association Declaration of Helsinki (2008). All students were invited at the start of their clinical orthodontic course and provided written informed consent prior to participation.

### Study population

The eligibility criteria were the provision of written consent and affiliation with a clinical orthodontic course. The exclusion criteria were long-term absence from the course, repetition of the course and the absence of signed informed consent.

All participants were undergraduate dental students enrolled in the state examination track (equivalent to a bachelor’s level) during their clinical orthodontic semesters. The learning content of the first clinical year (semesters seven and eight) covers essential aspects of orthodontic therapy, including preventive care and the collection of general and specific medical history. A key component is case evaluation through photo and model analyses, as well as the assessment of panoramic radiographs (OPGs) and lateral cephalometric radiographs (LCRs). Additionally, an overview of German orthodontic indication groups (KIGs) is provided. The participants will become familiar with various instruments and acquire practical skills in the fabrication and application of orthodontic appliances to patients.

The curriculum for the second clinical year (semesters nine and ten) offers an in-depth examination of essential elements in orthodontic diagnostics and treatment. The key focus areas include initial evaluations, diagnoses, and assessments of orthodontic indication groups. The covered topics encompass early intervention, trauma prevention, and trauma management. Furthermore, various therapeutic strategies addressing sagittal, vertical, and transverse malocclusions have been explored. Other crucial elements include the biology of tooth movement, multiple syndromes, cleft lip and palate, and biomechanical principles. The use of multibracket appliances, considerations for adult therapy, and challenges related to crowding and extraction treatment round out the curriculum. Students learned the theoretical and practical foundations of orthodontic case assessment through a combination of traditional lectures and specialised seminars, which were aligned with the university’s undergraduate orthodontic programme. All the students received the same instructional methods and resources, ensuring uniformity in information and teaching materials.

### Study design

**Fig. 1:**
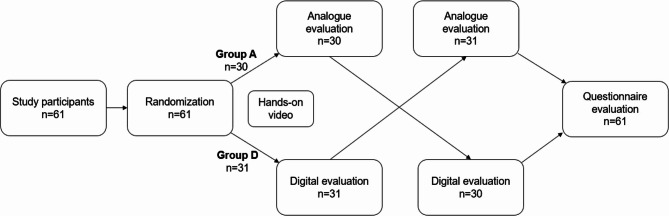
A randomised prospective crossover study: a total of 61 study participants were enrolled and randomly assigned to two groups: Group A (*n* = 30) and Group D (*n* = 31). Both groups initially received a standardised hands-on instructional video to ensure a uniform baseline of practical knowledge. Following the video, Group A first performed an analogue evaluation for 45 min, whereas Group D conducted a digital evaluation for the same duration. After the initial evaluation phase, the groups switched modalities: Group A then completed a digital evaluation, and Group D performed an analogue evaluation, each for 45 min. Upon completion of both evaluation tasks, all participants (*n* = 61) were asked to complete a standardised questionnaire assessing their experiences and perceptions regarding the digital evaluation

In this prospective crossover study (Fig. 1), the participants were randomly assigned to either group A (analogue) or group D (digital). Participants were randomized equally to the analogue-first (Group A) or digital-first (Group D) arm using a computer-generated list stratified by clinical year (first vs. second year). This approach ensured balanced representation of both in each study arm. Both groups were tasked with completing two orthodontic case planning exercises on the same day. In group A, case evaluation was initially performed analogically and subsequently digitised. Conversely, in group D, digital case planning was conducted first, and the analogue evaluation was subsequently performed. Time was stopped for each student once the exam was completed. The maximum time allowed for completion of each exam was 45 min. The selection of orthodontic cases for the digital and analogue mock exams comprised two patients exhibiting distal occlusion (Angle class II, division 2) of comparable extent at the molars and canines, accompanied by an overjet of over 6 mm and under 9 mm. A further requisite criterion entailed the presence of permanent dentition consisting of 28 erupted teeth and the existence of radiographic evidence of all fourth wisdom teeth. We conducted a quantitative validation of case equivalence using the Electronic Cast-Radiographic Evaluation (eCRE), which is based on the American Board of Orthodontics (ABO) Discrepancy Index. Initial models of both cases were scanned and analysed using digital ABO software, yielding almost identical difficulty scores and confirming comparable complexity. The validation was carried out on the software OnyxCeph.

Three experienced orthodontic experts created model solutions for the two orthodontic cases, and their results were averaged. For this purpose, both models were digitised via an intraoral scanner (3Shape Trios 3, 3Shape A/S, Copenhagen, Denmark) and analysed via the 3D software OnyxCeph (OnyxCeph^3TM^, Chemnitz, Germany), which uses the patch ‘Tooth with analysis Aachen’. The intraclass correlation coefficient between the three raters ranged from 0.931 to 0.990 and indicated excellent reliability.

The OPG and LCR were evaluated visually on a stationary PC with a diagnostic-quality screen, whereas the LCR was evaluated by marking reference points via the 3D software OnyxCeph (OnyxCeph^3TM^, Chemnitz, Germany).

### Conventional orthodontic case planning

In group A, orthodontic case planning was performed in the familiar setting of a traditional lecture hall. The participating students received a trimmed plaster model, a high-quality printed OPG, a LCR and a calliper (Munich model, Dentaurum, Ispringen, Germany). The students utilised the set square and dividers according to their own judgement. The results of their measurements were recorded on a printed evaluation sheet. The case solution was discussed in a follow-up appointment as part of the orthodontic seminar.

### Digital case planning

A web application named Orthotrainer was specifically created to educate and train orthodontic students. The application was created in reference to the printed evaluation form to perform an orthodontic diagnosis via digital means. The development was carried out in close cooperation with the Audiovisual Media Center, Aachen, to integrate modern technological and didactic approaches. The Orthotrainer is currently in use as a pilot version, restricted to students and staff at RWTH Aachen University for internal testing and feedback purposes. Once this validation phase is complete and any necessary refinements have been made, our plan is to release Orthotrainer as a freely accessible web tool for the wider dental education community. The Orthotrainer enables the clear presentation and interactive processing of complex content with a particular focus on assessing complex orthodontic diagnoses. To achieve this, multiple case studies are available within the Orthotrainer based on various KIG indications. Web-based implementation enables students to access and work on cases with internet-enabled devices, such as smartphones, tablets, PCs or laptops, which ensures flexible use of the application regardless of time and place. For this study, group D received a standard stationary computer, and orthodontic case planning was conducted via the Orthotrainer, which was designed in reference to the printed evaluation form to facilitate orthodontic diagnosis.

All the participating students were introduced to the Orthotrainer via a ten-minute practical video, which was distributed via the open-source learning platform Moodle (RWTHmoodle, RWTH Aachen University, Aachen, Germany), and subsequently shown on a projector in the lecture theatre prior to the digital examination. The video guided the study cohort step by step through the app and presented a sample case to ensure that the respective functions, applications and processes were understood.

**Fig. 2:**
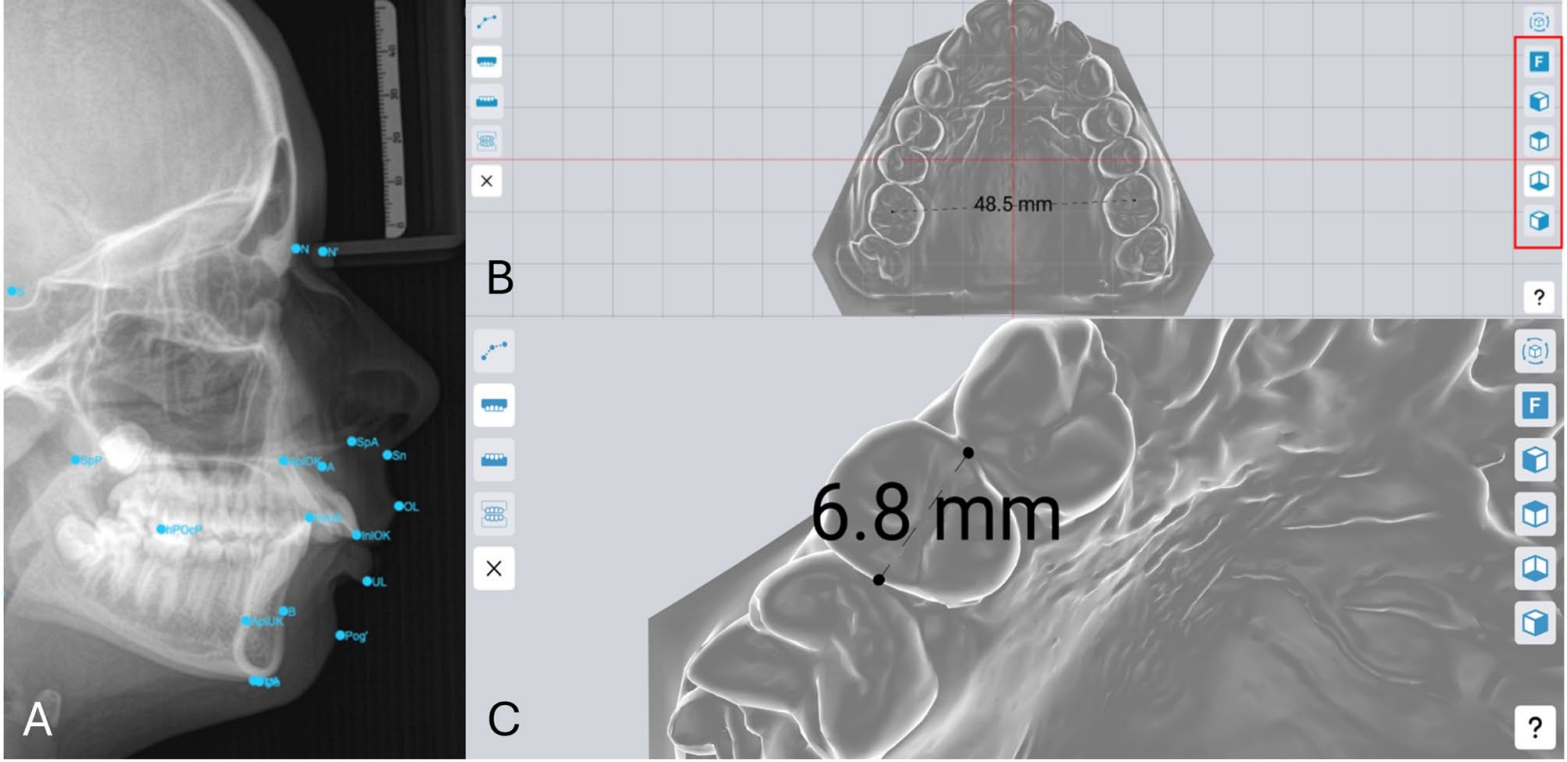
Illustration of the user interface of the e-learning application Orthotrainer Digital interface of cephalometric analysis: **A**. Each digital jaw can be displayed individually, rotated and enlarged via the zoom function **B**. The measurement function is enabled by the marking points **B**. Preset views of the 3D model enable a more precise evaluation of the model analysis **C**

The records to be examined in Orthotrainer included 3D study models that were digitised via an intraoral scanner, an OPG, and a LCR. Research has demonstrated the efficacy of this methodology, producing models with a high degree of accuracy and dimensions comparable to those of analogue plaster models [20, 21]. The participants were able to examine the records in accordance with classical orthodontic diagnostics, with the option to rotate and zoom in (Fig. 2). After completing each task, the students received immediate feedback within the app.

### Recording of orthodontic diagnostic performance

#### Orthodontic study models

The following parameters had to be assessed by the students in the analogue and digital study models:

1. Tooth size (15–25, 35–45) in millimeters [mm] was measured as the maximal mesiodistal width of the tooth (perpendicular to the tooth axis and parallel to the occlusal plane).
2. Anterior and posterior dental arch width of the maxilla in millimeters [mm]: distance between the deepest point of the fissure on the first premolar and the distance between the intersection of the anterior longitudinal fissure and the buccal transverse fissure of the six-year molars.
3. Anterior and posterior arch widths of the mandible in millimetres [mm]: distance between the contact point between the first and second premolar and distance between the tip of the mediobuccal cusp of the six-year molars.
4. Angle class: based on the relationship of the buccal groove of the mandibular first permanent molar and the mesiobuccal cusp of the maxillary first permanent molar [22].
5. Diagnosis of sagittal occlusion: static tooth pattern of the 1 st molar and canine in the sagittal plane.
6. Diagnosis of vertical discrepancies: static tooth pattern of 1 st molars, premolars and canines in the vertical plane.
7. Diagnosis of transverse deviations: static tooth pattern of 1 st molars, premolars and canines in the transverse plane.

#### Orthodontic X-ray diagnostics

The following parameters were recorded for the OPG as part of the radiological examination, if available (caries findings, fillings or bone resorption were not included in the assessment):

1. Tooth status and eruption.
2. Missing, extracted or supernumerary teeth.
3. Impacted teeth.
4. Root resorptions.

Each tooth was classified as ‘in occlusion’, ‘in active eruption’ or ‘unerupted’, with each correct classification earning one point. Overall success was calculated as the percentage of correctly classified teeth (number of correct classifications ÷ total assessable teeth × 100).

For the cephalometric analysis, the students were asked to correctly identify 15 hard tissue and five soft tissue reference points that the students were familiar with (Table 1). Reference points located within a radius of 2.00 mm were considered correct for performance analysis according to recent literature [23, 24]. Each student’s LCR success rate was then calculated as the percentage of correctly placed landmarks (number correct/15 × 100).

**Table 1:** Reference points of the cephalometric analysis: list of the 15 hard and 5 soft tissue points to be determined in the student cephalometric analysis

Point identification	
Hard tissue	
N - Nasion	Anterior end of the frontonasal sutura at the transition from the nasal to the frontal bone
S - Sella	Geometric centre of the Sella turcica in the median sagittal plain
ANS – Anterior nasal spine	Most anterior tip of the bony spina nasalis anterior (median sagittal plane)
PNS - Posterior nasal spine	The most posterior point of the nasal floor or bony palate in the extension of the anterior wall of the pterygopalatine fossa
A Point	The most dorsal point of the anterior contour of the maxillary alveolar process in the median-sagittal plane
B Point	The most dorsal point of the anterior contour of the mandibular alveolar process
Ar - Articulare	Radiological intersection between the dorsal contour of the ascending ramus and the lower edge of the skull base
Me - Menton	The most caudal point on the contour of the symphysis
Go (s) – Superior Gonion	The most dorsal point of the posterior margin of the ramus
Go (i) – Inferior Gonion	The most caudal point of the lower border of mandible
UIE – Upper incisor edge	Incisal edge of the most anterior upper central incisor
UIA – Upper incisor apex	Root tip of the most anterior upper central incisor
LIE – Lower incisor edge	incisal edge of the most anterior lower central incisor
LIA – Lower incisor apex	Root tip of the most anterior lower central incisor
PCOP – Posterior contactpoint of the occlusion planum	Most distal contact point of the first molars
Soft Tissue	
N´- Soft tissue nasion	Transition of the glabella to the bridge of the nose
Pog´- Soft tissue pogonion	Most protruding point of the chin
UL - Upper lip edge	The most anterior point of the upper lip
LL – Lower lip edge	The most anterior point of the lower lip
Sn – Subnasal	Transition between nose and upper lip

### Evaluation of the orthotrainer

After completing both orthodontic case planning exercises, all participants evaluated the app via a QR code via the online questionnaire tool SoSci Survey (SoSci Survey GmbH, Munich, Germany) (see Additional file 1). The following parameters were assessed:

1. Demographics: age, sex, semester
2. The Orthotrainer’s user-friendliness was assessed via the System Usability Scale (SUS), as described in previous studies [25, 26].
3. Performance of teaching methods and individual needs was assessed via the Online Learning Readiness Scale (OLRS) [27].
4. Students’ open-ended feedback and assessment.

### Statistical analysis

The data were recorded via Microsoft Excel (Office Version 365, Microsoft Corporation, Redmond, WA, USA) and transferred to GraphPad Prism (Version 10.0.1/2023, GraphPad Software, San Diego, CA, USA) for statistical analysis.

To increase the sample size of the present study, both orthodontic courses (first and second orthodontic years) were pooled. No formal power calculation was performed, as all 61 eligible dental students in the clinical orthodontic courses were enrolled. This cohort size was thus determined by full participation rather than by an a priori estimate. The Shapiro‒Wilk test was initially conducted to evaluate the normality of the data distribution. To assess students’ accuracy in the measurement parameters, deviations of the students’ values from the sample solution were compared via chi-square tests, Fisher’s exact test, or unpaired t tests.

The Shapiro‒Wilk test was initially conducted to evaluate the normality of the data distribution. An unpaired t test was used to determine whether there was a significant difference between groups in the overall evaluation of the LCR. Between-group differences in tooth size, dental arch width, sagittal dental occlusion, and individual point deviations in LCR (in millimeters) were compared via the unpaired t test or the Mann‒Whitney U test for nonnormally distributed data. To examine statistically significant associations between groups and parameters such as angle classification, transverse and vertical occlusion, and the correct selection of individual points in the LCR, Fisher’s exact test was applied. The level of significance was set at α = 0.05 for all the statistical tests.

## Results

All sixty-one students provided written consent, completed both evaluation modalities, and were included in the analyses; no dropouts or missing data occurred. Sixty-one dental students (47 females, 14 males, mean age: 25.8 ± 4.3 years) attending clinical orthodontic courses participated in this prospective comparative study at the medical faculty of RWTH Aachen University. Twenty-two students (mean age: 24.8 ± 4.6 years) were in their first clinical year of orthodontics (7th and 8th semester), and thirty-nine (mean age: 26.3 ± 4.0 years) were in their second clinical year (9th and 10th semester). The Orthotrainer demonstrated that the technical implementation of comprehensive 3D analysis of complex anatomical structures is feasible and effective. 3D evaluations using digital models, orthopantomograms (OPGs), and lateral cephalometric radiographs (LCRs) can be successfully performed and are comparable in performance to traditional analogue methods. The mean duration for completing digital case planning was 26.3 ± 6.4 min, whereas analogue planning required 25.4 ± 6.5 min, with no significant difference between the two approaches (*p* > 0.05). To evaluate the equivalence of the diagnostic performance of analogue and digital methods, and to assess user acceptance, our results are presented in three consecutive sections. First, we present the measurements from the model analysis. Secondly, the OPG and LCR results are described in detail in the radiological evaluation. Finally, the results of the Online Learning Readiness Scale (OLRS) and the System Usability Scale (SUS), as well as the open feedback round, are summarised in the evaluation and user feedback section.

### Model analysis performance

#### Tooth width measurements

The mean measurement discrepancies for the individual tooth widths resulted in variations of 0.35–0.63 mm for group D in the upper jaw (teeth 15–25) and 0.32–0.82 mm for group A in the lower jaw (Table 2). Similarly, satisfactory measurements were obtained, with mean individual deviations of 0.30–0.49 mm for group D and 0.22–0.43 mm for group A. No statistically significant differences were observed between the groups in the measurement of tooth width (*p* > 0.05) (Table 2).

**Table 2:** Tooth width analysis:the results of deviations from the model solution in determining the widths of teeth 15–25 and 35–45, respectively, as determined by students via analogue and digital (Orthotrainer application) methods. SD = standard deviation, sem = standard error, ns = no statistically significant difference

		analogue						digital						
tooth	n	∆ mean (mm)	SD	SEM	Lower 95% CI	Upper 95% CI	n	∆ mean (mm)	SD	SEM	Lower 95% CI	Upper 95% CI	p value	
15	61	0.543	1.127	0.144	0.254	0.831	61	0.434	0.535	0.069	0.297	0.572	0.998	ns
14	61	0.500	0.583	0.075	0.351	0.649	61	0.400	0.468	0.060	0.280	0.520	0.999	ns
13	61	0.512	0.747	0.096	0.320	0.703	61	0.331	0.318	0.041	0.250	0.413	0.919	ns
12	61	0.380	0.748	0.096	0.189	0.572	61	0.582	1.069	0.137	0.308	0.856	0.849	ns
11	61	0.490	0.640	0.082	0.326	0.654	61	0.820	1.490	0.191	0.438	1.201	0.230	ns
21	61	0.490	0.633	0.081	0.328	0.652	61	0.693	1.686	0.216	0.262	1.125	0.842	ns
22	61	0.348	0.617	0.079	0.189	0.506	61	0.633	1.035	0.133	0.368	0.898	0.423	ns
23	61	0.490	0.607	0.078	0.335	0.646	61	0.351	0.383	0.049	0.253	0.449	0.986	ns
24	61	0.629	0.691	0.088	0.452	0.806	61	0.441	0.285	0.037	0.368	0.514	0.897	ns
25	61	0.442	0.593	0.076	0.290	0.594	61	0.320	0.233	0.030	0.260	0.379	0.995	ns
35	61	0.433	0.592	0.076	0.281	0.584	61	0.425	0.503	0.064	0.296	0.553	> 0.999	ns
34	61	0.426	0.618	0.079	0.268	0.585	61	0.290	0.210	0.027	0.237	0.344	0.714	ns
33	61	0.480	0.677	0.087	0.307	0.654	61	0.328	0.350	0.045	0.238	0.418	0.563	ns
32	61	0.451	0.588	0.075	0.300	0.601	61	0.215	0.177	0.023	0.169	0.260	0.065	ns
31	61	0.315	0.650	0.083	0.148	0.481	61	0.234	0.200	0.026	0.183	0.286	0.988	ns
41	61	0.303	0.595	0.076	0.151	0.456	61	0.308	0.242	0.031	0.246	0.370	> 0.999	ns
42	61	0.382	0.603	0.077	0.228	0.536	61	0.279	0.221	0.028	0.222	0.335	0.931	ns
43	61	0.493	0.689	0.088	0.317	0.670	61	0.362	0.401	0.051	0.260	0.465	0.755	ns
44	61	0.451	0.567	0.073	0.306	0.596	61	0.264	0.257	0.033	0.198	0.330	0.275	ns
45	61	0.484	0.504	0.064	0.355	0.613	61	0.241	0.222	0.028	0.184	0.298	0.052	ns

*L 95% CI* Lower 95% CI of the mean

#### Dental arch width measurements

**Fig. 3:**
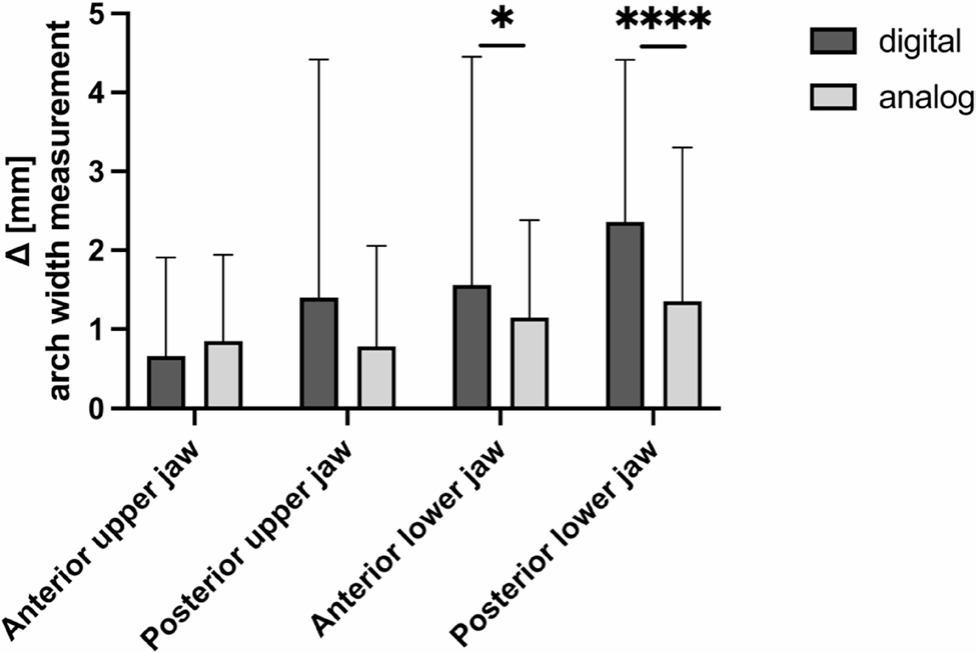
Results of the dental arch width measurements: mean deviations from the model solution (Δ) regarding dental arch width (mm) determined with the digital (Orthotrainer application, dark gray) and analogue (light gray) methods in the anterior/posterior maxilla and the anterior/posterior mandible; statistically significant differences are marked with *p < 0.05, ****p < 0.0001; mean ± SD

The analysis of the width of the dental arch in the upper jaw did not reveal any significant differences in either the anterior (*p* = 0.364) or posterior (*p* = 0.988) regions (Fig. 3; Table 3). In the mandible, significant differences were found in favour of the analogue measurement method, with an average deviation of 1.15 ± 1.23 mm in group A compared with 1.56 ± 2.89 mm in group D (*p* = 0.0137) and an average deviation of 1.36 ± 1.95 mm in group A and 2.36 ± 2.05 mm in group D (*p* < 0.0001) (Fig. 3; Table 3).

**Table 3:** Anterior and posterior arch width analysis: the results of deviations from the model solution in determining the upper and lower anterior and posterior arch width. UJ = upper jaw, lw = lower jaw, sd = standard deviation, sem = standard error, ns = no statistically significant difference; statistically significant differences are marked with p*≤0.05, p****<0.0001

		analogue						digital						
	n	∆ mean (mm)	SD	SEM	Lower 95% CI	Upper 95% CI	n	∆ mean (mm)	SD	SEM	Lower 95% CI	Upper 95% CI	p value	
anterior UJ	61	0.855	1.088	0.140	0.070	0.930	61	0.665	1.242	0.160	0.170	0.470	0.364	ns
anterior LJ	61	1.149	1.234	0.161	0.600	0.900	61	1.563	2.888	0.376	0.230	0.630	0.0137	*
posterior UJ	61	0.787	1.269	0.164	0.500	0.500	61	1.402	3.016	0.389	0.400	0.600	0.988	ns
posterior LJ	61	1.356	1.947	0.254	0.330	0.670	61	2.362	2.054	0.265	1.130	2.430	< 0.0001	****

*L 95% CI* Lower 95% CI of the mean, *UJ* upper jaw, *LJ* lower jaw

#### Analysis of the occlusion

**Fig. 4:**
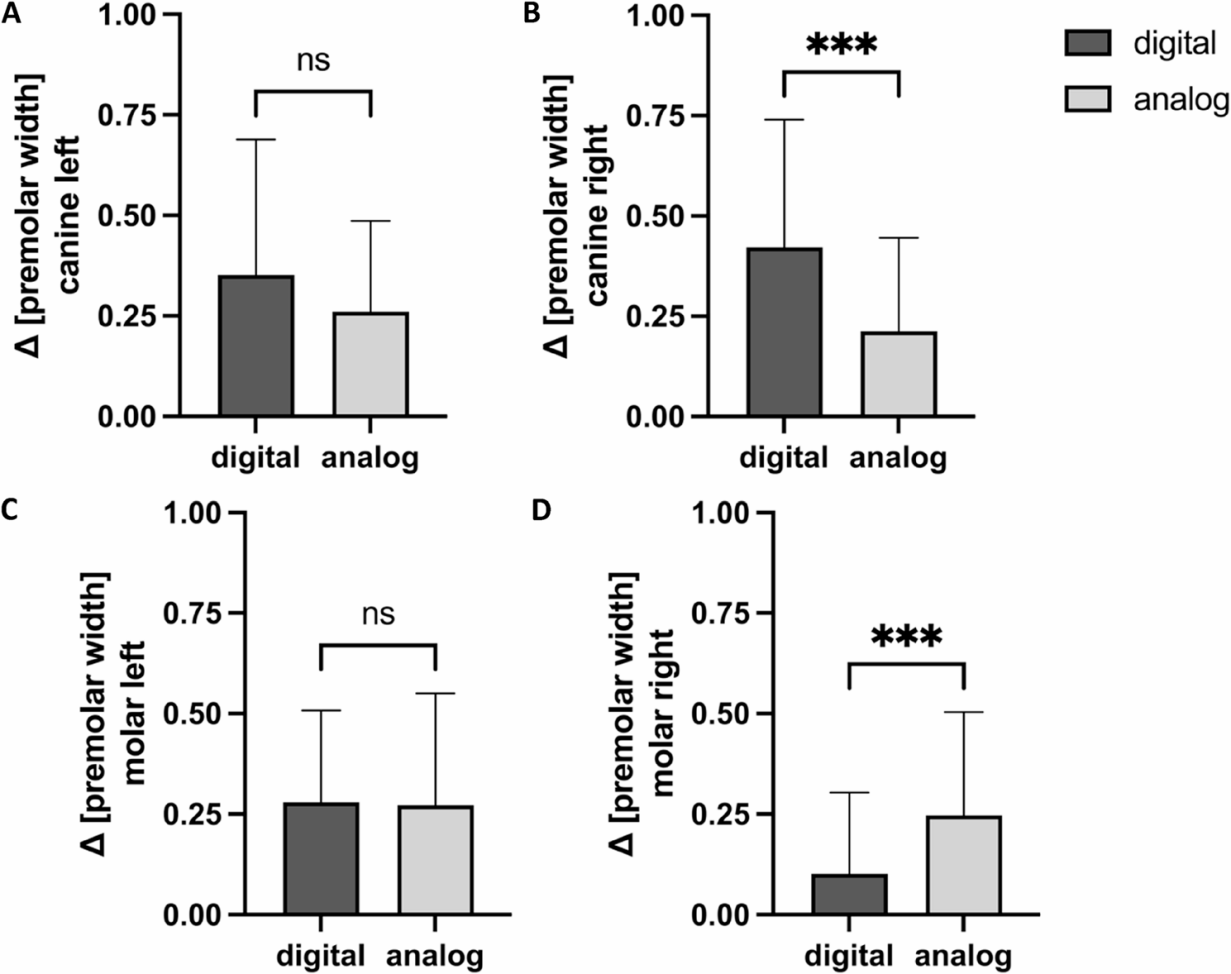
Determining the Angle class: deviations of the cohort groups from the model solution in the molar and canine region in a right/left comparison [in pw]; sagittal deviation of the canine left (**A**), canine right (**B**), molar left (**C**) and molar right (**D**); statistically significant differences are marked with ***p < 0.001; mean ± SD

To determine the intermaxillary deviation of the sagittal occlusion, group D determined the Angle class on the right side of the molar, with an average deviation of 0.10 ± 0.20 premolar widths (pw) greater than that of group A, with 0.25 ± 0.26 pw (*p* = 0.0003). The canine on the right was determined to have a greater deviation from group D by 0.42 ± 0.32 pw than from group A, with a 0.21 ± 0.23 pw deviation (*p* = 0.0001). The left-sided intermaxillary analysis revealed the sagittal relationships for molars (*p* = 0.607) and canines (*p* = 0.166) equally well for both groups (molar left: group D 0.28 ± 0.23 pw, group A 0.27 ± 0.28 pw; canine left: group D 0.35 ± 0.34 pw, group A 0.26 ± 0.23 pw), with no significant differences (Fig. 4).

The intermaxillary analysis of the vertical and transverse deviations demonstrated a strong correlation between the students and the sample solution, with percentages reaching up to 96.72% in group D and 93.44% in group A, with no significant differences (*p* > 0.05). The intermaxillary analysis of the vertical and transverse deviations of the canines demonstrated a sustained yet marginally diminished correlation between the students and the sample solution, with percentages reaching 91.80% in group D and 85.25% in group A, with no significant differences (*p* > 0.05) (Table 4).

**Table 4:** Analysis of the occlusion: student success rates in determining sagittal, vertical and transverse occlusion; ns = no statistically significant difference

	*n*	digital success rate	*n*	analogue success rate	*p* value	
Angle-Class	61	86.89%	61	83.61%	0.7992	ns
Vertical relation of the right molars	61	95.08%	61	90.16%	0.4909	ns
Vertical relation of the left molars	61	91.80%	61	91.80%	> 0.9999	ns
Vertical relation of the right premolars	61	95.08%	61	91.80%	0.7172	ns
Vertical relation of the left premolars	61	95.08%	61	93.44%	> 0.9999	ns
Vertical relation of the right canine	61	91.80%	61	78.69%	0.0718	ns
Vertical relation of the left canine	61	90.16%	61	83.61%	0.4219	ns
Transversal relation of the right molars	61	91.80%	61	93.44%	> 0.9999	ns
Transversal relation of the left molars	61	95.08%	61	91.80%	0.7172	ns
Transversal relation of the right premolars	61	96.72%	61	90.16%	0.2724	ns
Transversal relation of the left premolars	61	51.72%	61	48.28%	0.2070	ns
Transversal relation of the right canine	61	91.80%	61	78.69%	0.0718	ns
Transversal relation of the left canine	61	88.52%	61	85.25%	0.7894	ns

### Outcome of the radiographic evaluation

#### Evaluation of the orthopantomogram

Group D achieved a success rate of 97.09% for the OPG, and group A achieved a success rate of 95.64% when only the patient’s dental status was used.

#### Cephalometric analysis

**Fig. 5:**
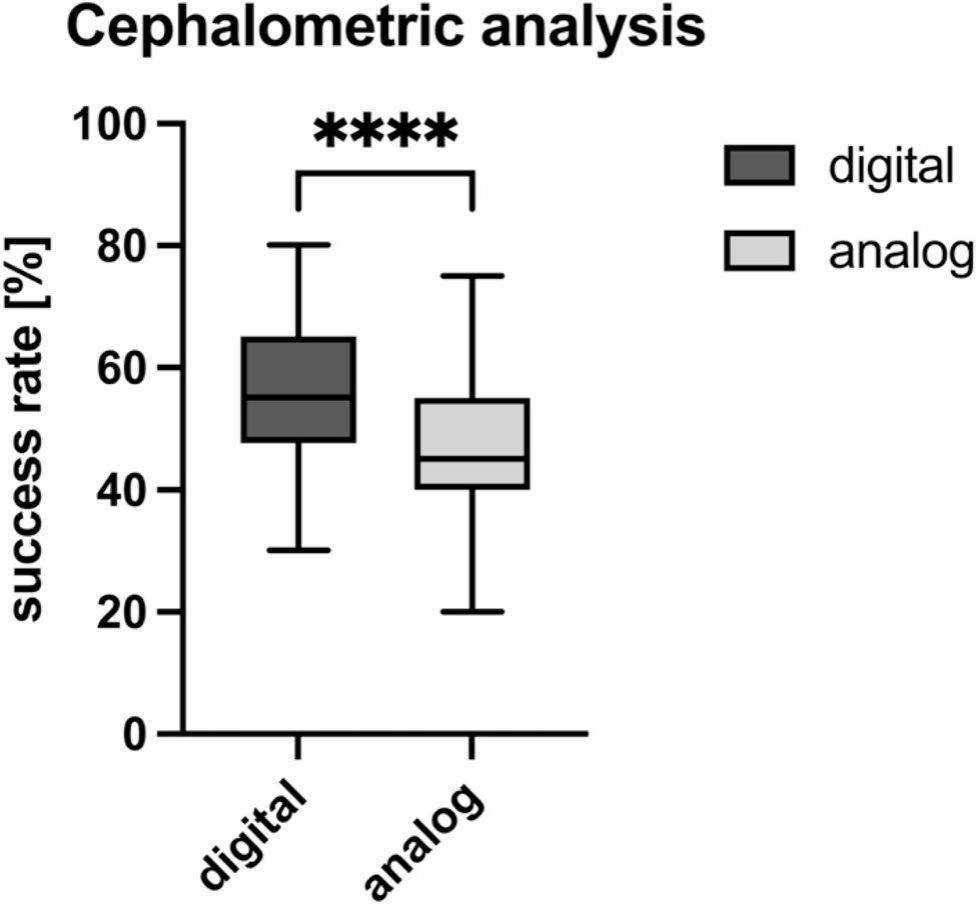
Success rate of the cephalometric analysis: success rate for determining cephalometric hard and soft tissue points between the groups; statistically significant differences are marked with ****p < 0.0001; mean ± SD

The results revealed that, compared with group A, group D yielded more precise results in analysing the LCRs, with a mean deviation of 3.03 ± 1.97 mm from the correct values, with a mean deviation of 4.12 ± 3.37 mm (Table 5). Similarly, group D reached a significantly higher overall success rate than did group A (55.74 ± 11.06 vs. 46.80 ± 11.69%; *p* < 0.0001; Fig. 5).

**Table 5:** Cephalometric analysis: the results of deviations in the cephalometric evaluation of both groups [in mm]. SD = standard deviation, sem = standard error, ns = no statistically significant difference; statistically significant differences are marked with p*≤0.05, p**<0.01, p***<0.001, p****<0.0001

		analogue						digital						
Point	n	∆ mean (mm)	SD	SEM	Lower 95% CI	Upper 95% CI	n	∆ mean (mm)	SD	SEM	Lower 95% CI	Upper 95% CI	p value	
N	61	2.108	3.271	0.419	1.271	2.946	61	3.505	2.838	0.363	2.778	4.232	< 0.0001	****
S	61	1.568	0.468	0.060	1.447	1.689	61	0.530	0.507	0.065	0.400	0.660	< 0.0001	****
ANS	61	7.581	5.103	0.664	6.251	8.911	61	5.461	5.908	0.756	3.948	6.974	0.0083	**
PNS	61	6.108	3.223	0.416	5.276	6.941	61	5.770	5.702	0.730	4.310	7.231	0.1516	ns
A	61	5.223	2.977	0.384	4.454	5.992	61	1.521	2.811	0.360	0.801	2.241	< 0.0001	****
B	61	1.992	1.341	0.172	1.648	2.335	61	2.392	1.193	0.153	2.086	2.697	0.0105	*
Me	61	5.139	3.691	0.473	4.194	6.085	61	4.211	4.466	0.572	3.068	5.355	0.0115	*
Ar	61	5.423	6.500	0.832	3.758	7.088	61	6.883	6.586	0.850	5.182	8.585	0.6350	ns
Go(i)	61	3.370	1.895	0.2446	2.880	3.860	61	5.593	11.000	1.408	2.777	8.410	0.7594	ns
Go(s)	61	5.733	7.994	1.023	3.686	7.780	61	7.598	12.750	1.646	4.305	10.980	0.6461	ns
UIA	61	4.142	1.450	0.187	3.767	4.516	61	1.466	2.552	0.327	0.812	2.119	< 0.0001	****
UIE	61	0.963	0.788	0.101	0.762	1.166	61	0.371	0.528	0.0676	0.235	0.506	< 0.0001	****
LIE	61	0.807	0.597	0.0764	0.654	0.959	61	0.305	0.424	0.0542	0.196	0.413	< 0.0001	****
LIA	61	2.420	1.165	0.149	2.121	2.718	61	1.564	0.882	0.113	1.338	1.790	< 0.0001	****
PCOP	61	6.802	5.574	0.7319	5.336	8.267	61	8.210	8.156	1.044	6.121	10.300	0.4683	ns
N`	61	2.128	1.150	0.149	1.831	2.425	61	2.969	5.778	0.740	1.489	4.449	0.6384	ns
Pog`	61	2.476	2.563	0.334	1.808	3.144	61	2.921	3.774	0.4832	1.955	3.888	0.2152	ns
UL	61	1.448	0.622	0.080	1.288	1.607	61	1.305	1.216	0.156	0.994	1.616	0.0218	*
LL	61	2.687	1.259	0.161	2.364	3.009	61	3.116	1.640	0.210	2.696	3.536	0.0663	ns
Sn	61	1.287	0.652	0.083	1.120	1.454	61	2.339	1.174	0.150	2.039	2.640	< 0.0001	****

However, the aggregate analysis indicated that the digital method demonstrated enhanced reliability in determining the reference points. Major differences were observed in the reference point of the apex of the upper incisor (UIA), which was successfully detected in 88.52% of the students in group A and in only 1.64% of those in group D (*p* < 0.0001). Although some points were marked with less success in the digital analysis, the success rate was never below 11.48% (Table 6).

**Table 6:** Success rate in determining cephalometric reference points: the comparison of the success rates in determining cephalometric reference points indicates that specific points were difficult for the students to set in the analogue and digital evaluation methods. The differences mainly concerned the hard tissue points, with only partial overlaps between both groups; ns=no statistically significant difference; statistically significant differences are marked with p*≤0.05, p**<0.01, p***<0.001, p****<0.0001

Point	*n*	digital success rate	analogue success rate	*p* value	
S	61	100.00%	86.89%	0.0061	**
UIE	61	98.36%	90.16%	0.1142	ns
LIE	61	98.36%	100.00%	> 0.9999	ns
UIA	61	88.52%	1.64%	< 0.0001	****
UL	61	86.89%	86.89%	> 0.9999	ns
A	61	80.33%	18.03%	< 0.0001	****
Pog`	61	65.57%	50.82%	0.1417	ns
LIA	61	63.93%	39.34%	0.0109	*
N`	61	57.38%	47.54%	0.3648	ns
Me	61	45.90%	18.03%	0.0017	**
ANS	61	45.90%	16.39%	0.0008	***
PNS	61	44.26%	18.03%	0.0031	**
N	61	39.34%	77.05%	< 0.0001	****
Go(s)	61	36.07%	21.31%	0.1086	ns
B	61	34.43%	65.57%	0.0010	**
Sn	61	32.79%	91.80%	< 0.0001	****
PCOP	61	31.15%	32.79%	> 0.9999	ns
LL	61	27.87%	34.43%	0.5579	ns
Ar	61	24.59%	21.31%	0.8298	ns
Go(i)	61	11.48%	18.03%	0.6461	ns

### Evaluation of the orthotrainer and user feedback

#### Online learning readiness scale (OLRS)

**Fig. 6:**
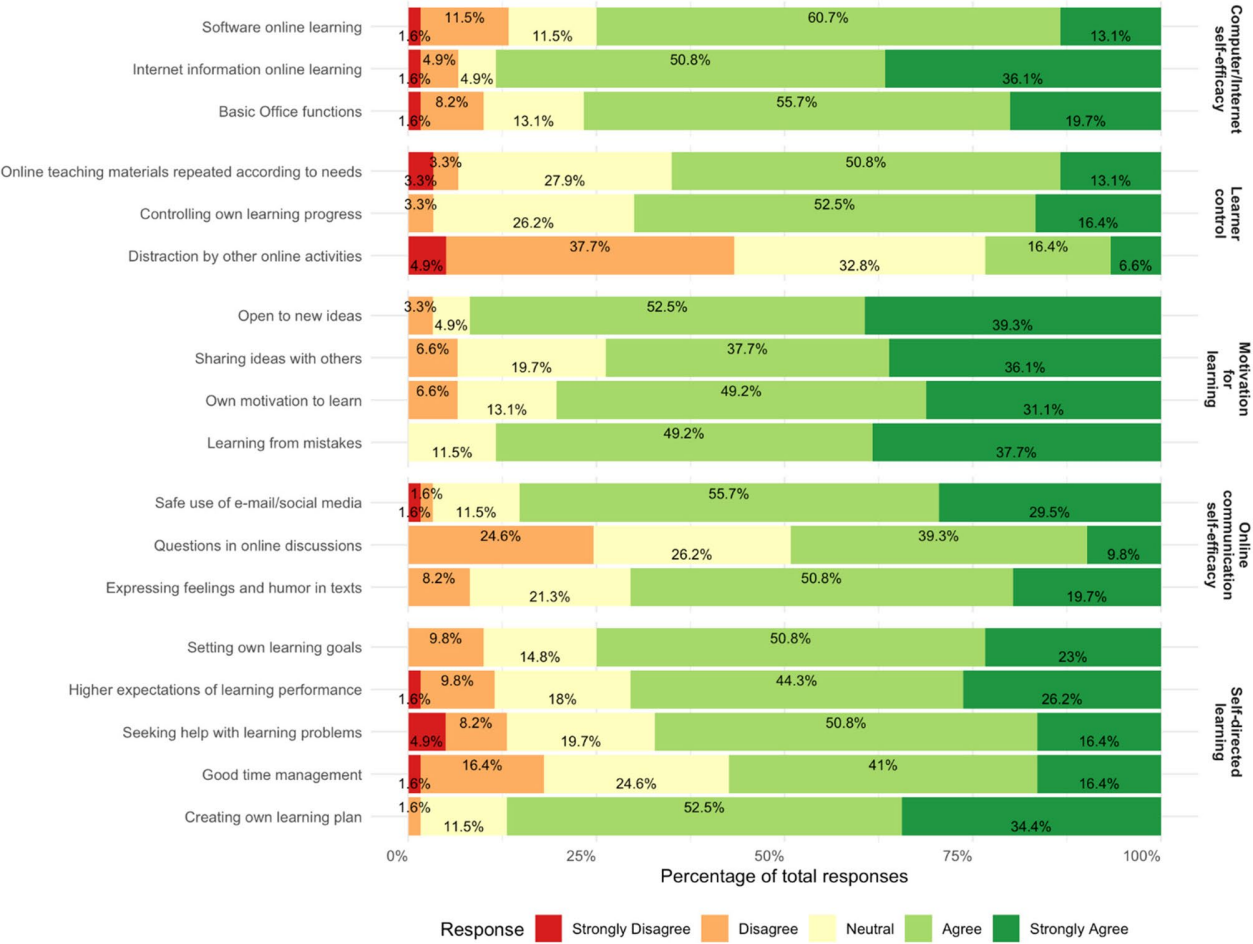
Results of the learner readiness for online learning questionnaire: the following figure illustrates a percentage distribution of responses related to varying dimensions of online learning readiness. The categories include computer/Internet self-efficacy, learner control, motivation for learning, online communication self-efficacy and self-directed learning. Most participants showed high agreement (agree, strongly agree) in all dimensions except ‘distraction by other online activities’, whereas the ‘learner control’ dimension received the lowest relative agreement

Following the completion of both practice rounds, all 61 students participated in the survey (completion rate: 100%). A descriptive statistical analysis was conducted to ascertain the students’ self-assessment of their level of preparation to undertake a digital course (Fig. 6). The categories include computer/Internet self-efficacy (mean 3.92 ± 0.87), learner control (mean 3.44 ± 0.98), motivation for learning (mean 4.16 ± 0.79), online communication self-efficacy (mean 3.75 ± 0.92) and self-directed learning (mean 3.82 ± 0.95). This analysis was facilitated by the utilisation of bar graphs, which enabled the data to be systematically interpreted. The results indicate that 73.8% of the students were confident in learning software (60.7% agreed; 13.1% strongly agreed), and 70.5% were proficient in utilising internet information (50.8% agreed; 19.7% strongly agreed). This high level of technological competence is crucial for success in a digital learning environment.

With respect to learner control, more than half of the students demonstrated strong self-regulation capabilities, with a combined agreement rate of 65.6% (52.5% agree; 13.1% strongly agree) concerning managing their own learning progress and repeating materials as needed.

The motivation for learning is also pronounced: the majority of the students (91.8%) are open to new ideas (52.5% agree; 39.3% strongly agree) and enjoy sharing knowledge (total 73.8%; 37.7% agree; 36.1% strongly agree). While most students feel ready to safely use email and social media (total: 85.2%; 55.7% agree; 29.5% strongly agree), only 49.1% feel prepared to engage in online discussions (39.3% agree; 9.8% strongly agree).

The ability for self-directed learning is supported by positive attitudes toward setting personal goals (total 73.8%, 50.8% agree, 23% strongly agree) as well as maintaining high performance expectations (total 70.5%; 44.3% agree; 26.2% strongly agree).

#### System usability scale (SUS)

**Fig. 7:**
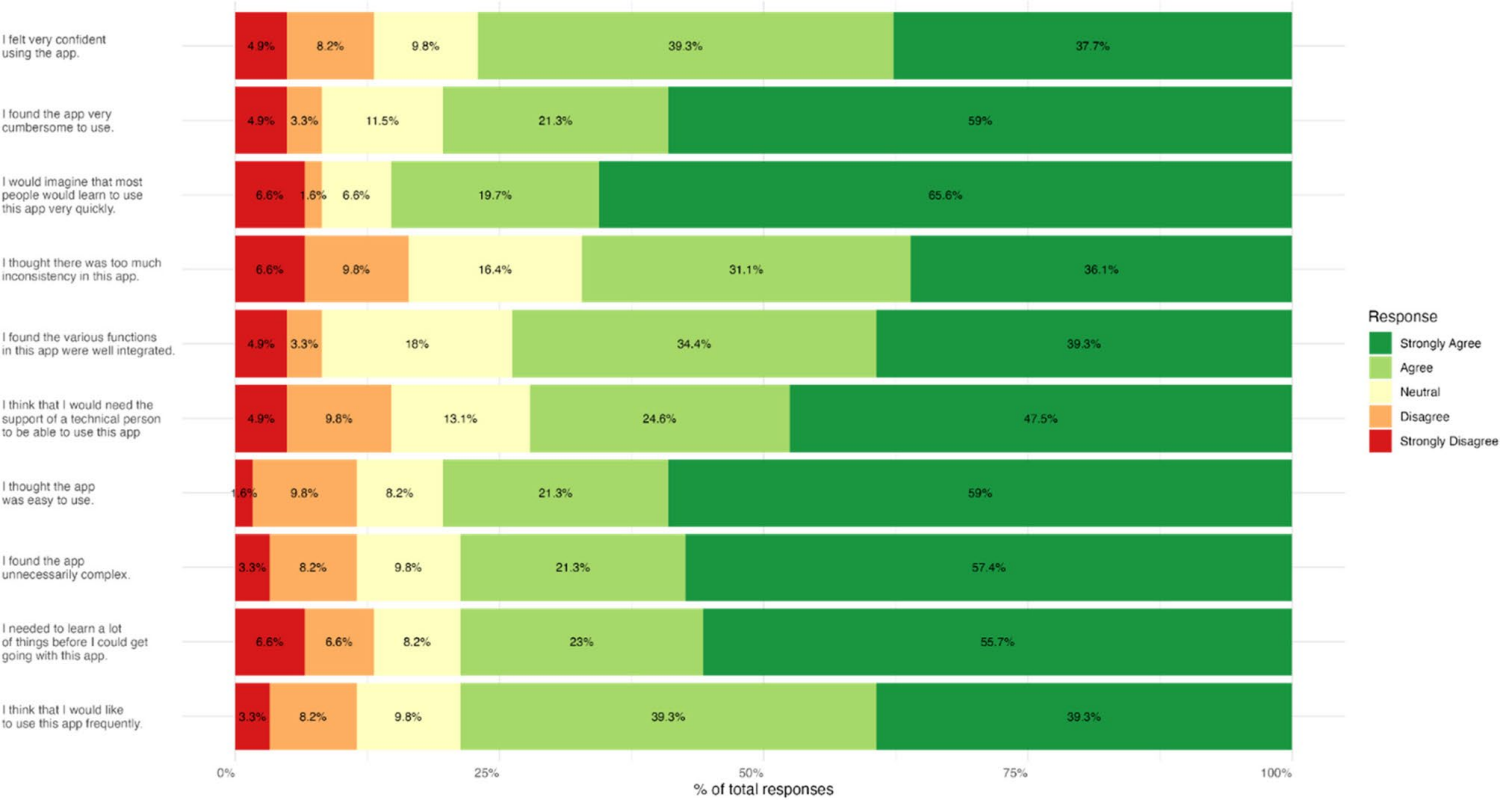
Results of the System Usability Scale (SUS): the following figure illustrates the percentage distribution of responses to different aspects of an application’s usability. The scale ranges from ‘strongly disagree’ (red) to ‘strongly agree’ (dark green). While most participants were positive about the application (‘strongly agree’ or ‘agree’), only 39.3% strongly agreed that they ‘would like to use the application frequently’. These results suggest that there is room for improvement, particularly in terms of long-term user retention

The mean SUS score was 77.62 (SD: 23.16; 95% confidence interval: 71.70–83.56), indicating good usability of the application (Fig. 7). While most participants expressed positive sentiment toward the application (“strongly agree” or “agree”), only 39.3% strongly agreed that they “would like to use the app frequently”.

#### Open feedback of the students

The participants in the study particularly emphasised the ease and intuitive use of the Orthotrainer. The software appeared to be quick to learn and efficient to use, making the digital learning process perceived as time-saving and clear. Smooth navigation, the ability to enter values directly without constant scrolling, and clear visualisation through animations and the grid diagram were particularly desirable. Digital measurement also made the process easier than manual methods using rulers or callipers. The ability to move models and analyse tooth structures interactively was considered an advantage. However, some areas for improvement were identified. In particular, the accuracy of the measurements was criticised, especially when reference points were assessed in the cephalometric analysis and in the tooth width measurements. The control of mouse movements and zooming was also unintuitive in some cases. Some users wished for greater precision in model control and the ability to manually correct certain measurements. Notably, data from previous runs should not be automatically saved to avoid unwanted influences. The participants rated the software with an average score of 1.56. In the German rating system, a score of 1 corresponds to the best rating.

## Discussion

Diagnostic measurements performed with Orthotrainer were comparable to analogue methods for tooth and arch width, with superior precision in cephalometric landmark identification. These findings confirm that web-based tools can match traditional workflows in case planning.

### Diagnostic reliability of the digital method compared to the analogue method

For arch width analysis in the lower jaw, minimally better measurements were found in favour of the analogue measurement. In the lower jaw, where reference points are set to contact points, this is not always clearly recognizable in the digital study model. Furthermore, the buccal cusp is more challenging to identify in the lower jaw than a central fissure in the upper jaw. Consequently, it is recommended that the measurement of arch width in the lower jaw be incorporated into future exercises in the Orthotrainer. The deviations were therefore clinically acceptable.

The present study demonstrated that both cohorts accurately identified intermaxillary deviations with high success rates, but the differences were not significant. Only when the classification of the angle class was considered were there significant differences in the determination of occlusion on the right side. This was probably because although the digital and analogue exercises were identical in terms of difficulty, there was no deviation of a full premolar width in the analogue case in the molar region, and this had to be determined. It seems that the students found it difficult to determine the occlusion using both methods, so further exercises should be carried out in the future.

The OPG was evaluated, and comparable performance was demonstrated by both groups on the basis of their dental status. Here, the students show some difficulty in determining whether the 2nd molar (17, 27, 37, 47) is fully erupted, resulting in a small loss in the success rate.

The results of the present study show that, compared with group A, group D demonstrated a significantly greater success rate in cephalometric analysis and, in most cases, a significantly more precise determination of the reference points. This substantial difference suggests that the e-learning platform may be particularly effective in teaching the complex analytical skills required for cephalometric evaluation. With respect to the individual landmarks, not all of them could be identified by the students with complete satisfaction, a problem partly due to the presentation of cephalometrics, which also plays a role in everyday clinical practice. Cephalometry involves depicting a three-dimensional object in two dimensions, and the process of identifying points on a radiograph relies on both existing anatomical landmarks and constructed landmarks (e.g., sella). In instances where two outlines of bilaterally existing points are present, a “mean” landmark is used as a compromise. The reliability of identifying different landmarks is subject to variation, as identification is based on contrast to other surrounding structures on the radiograph [28, 29]. Several challenges arise in the reliable identification of structures, such as the apex of the incisors, which exhibit suboptimal repeatability and reproducibility. The comprehensive evaluation of landmark identification of hard and soft tissue points in favour of group D indicates that students can competently execute a digital cephalometric lateral analysis without a significant preparation period.

### Usability and self-directed learning

The orthotrainer’s ease of use and intuitiveness were highly valued by the study participants. The digital learning method was seen as time-saving and simple since the software was easy to use and quick to learn, although minor adjustments to the control system and measurement accuracy could contribute to further improvements.

Beyond diagnostic performance, the survey data underscore Orthotrainer’s potential to foster flexible, accessible, and self-directed learning. High OLRS domain scores—particularly in computer/Internet self-efficacy and learning motivation—demonstrate that students feel confident navigating the platform independently and are intrinsically motivated to engage with its case modules. Open-ended feedback further highlighted the convenience of accessing cases anytime, the ability to repeat measurements at one’s own pace, and the value of instant feedback. These features align with adult learning principles and suggest that Orthotrainer can complement traditional lectures by enabling students to practice and refine diagnostic skills outside scheduled class hours, thereby enhancing learner autonomy, engagement, and satisfaction.

An SUS score of 77.62 is above average and is often categorised as ‘good’ or just below ‘excellent’, which indicates a high level of user-friendliness. In other words, the Orthotrainer was perceived as considerably more usable than the benchmarks, which were based on hundreds of systems and thousands of individual SUS ratings. The average SUS score was 68 [30, 31]. The benchmark of 80 was narrowly missed, which means a rating of A- in the Sauro-Lewis rating system. The comparison of e-learning products is inherently constrained by their heterogeneity in design and target audience; however, it is important to note that the system usability scale (SUS) serves as the established standard specifically developed and validated for the evaluation and comparison of the usability of digital products [26, 30]. With an average SUS score of 77.62, the Orthotrainer app received a rating of ‘B+’ according to Sauro and Lewis [30, 31]. According to Bangor et al., this can be characterised as ‘good’ [32] and is therefore rated as ‘acceptable’ [33]. These findings indicate good acceptance of the method but also highlight potential areas for improvement, particularly in sustaining long-term user engagement.

However, there are a few limitations to note. First, it should be noted that the clinical treatment courses in the Department of Orthodontics are practiced and discussed entirely in analogue form, meaning that group A had a significantly better starting point than did group D in terms of approach and handling. However, to that end, a 10-minute practical video introduced the Orthotrainer so that the students could familiarise themselves with the new tool to minimise the familiarity effect of the traditional format.

Second, it is important not to disregard clear limitations, such as the fact that the data were collected at only one dental school. Students from other universities who have had different online experiences may react differently.

Third, to increase the number of participants and gain a substantive impression of the app’s applicability, students with different levels of knowledge were included in the study. The randomisation of pooled study cohorts across different semesters could introduce systematic biases that impact the results. Panel data analyses highlight semester-related confounders such as varying examination conditions and workload [34, 35]. For example, stricter exam modalities could influence group D performance independent of e-learning performance. Additionally, time effects, such as “end-of-semester syndrome,” may skew success rates if a digital cohort’s main learning phase coincides with peak academic stress. However, in this study, both semesters were evenly distributed between groups, test runs were conducted on the same day, and usability was the primary focus, mitigating bias. While digital evaluation is not a core component of the orthodontic curriculum, external learning influences could still affect usability outcomes, necessitating cautious interpretation [9, 35]. A more precise analysis should be carried out in the future to determine which students experience a particularly high increase in knowledge through the app. This allows for the integration of the app into orthodontic courses as effectively as possible and ensures the achievement of the best possible results in the final orthodontic examination.

### Future directions

There is an absence of studies in the literature that have utilised a web-based application with macroactive content for the purposes of orthodontic teaching in diagnostics and case evaluation and that have compared it with traditional analogue learning content. Therefore, it may be beneficial to repeat the present study with a larger sample size, allowing for a more detailed analysis that ideally incorporates sophisticated qualitative and quantitative methods. Future research should use robust study designs – such as randomised controlled trials with pre- and post-tests and longitudinal follow-ups at 6- and 12-month intervals – to quantify the actual learning gains and knowledge acquisition attributable to Orthotrainers. Multicentre collaborations will improve external validity, while integrating the platform into existing seminar plans will enable the assessment of optimal timing, frequency and resource implications. Objective outcome measures (e.g., case planning tests, accuracy of cephalometric analysis) should be supplemented by qualitative feedback (focus groups, teacher surveys) to identify barriers and facilitating factors and thus determine best practices for seamless integration into the curriculum.

In order to facilitate practical implementation in the context of university life, the creation of a comprehensive instructional manual could be considered, in addition to the video, with a view to enabling students to outsource preparation time if necessary. The 10-minute tutorial was found to be adequate for inexperienced users in the study while concurrently constituting a minimal investment in training. To facilitate the integration of these resources into existing curricula, the involvement of a subject matter expert in the form of dedicated sessions may be advantageous in order to ensure the full acclimatisation of students and the answering of any remaining questions. The administration of follow-up surveys to students and physicians in a qualitative manner has the potential to yield value to optimise the implementation. The web-based nature of the Orthotrainer minimises hardware requirements, as it is compatible with any internet-enabled device. However, it is imperative that facilities ensure the provision. of reliable network access. It is recommended that subsequent cost-benefit analyses make a comparison between the financial implications of hosting and maintaining the software and the economic benefits of reducing expenditure on the production of physical models and teacher support time.

Overall, the Orthotrainer provides diagnostic performance comparable to analogue methods for tooth and arch measurements. It achieves a significantly higher success rate in identifying lateral cephalometric landmarks and receives high usability scores. The combination of accurate measurements, proficiency in radiography, and a positive user experience suggests that Orthotrainer not only replicates traditional workflows, but also offers unique advantages for radiographic training by providing immediate, interactive feedback that has the potential to reinforce learning and boost confidence in landmark placement.

In conclusion, digital learning platforms such as Orthotrainer have the potential to fundamentally change orthodontic training and practice. These platforms present novel prospects for effective learning, practical exercises and continuous professional development. The challenge, however, lies in the further development and integration of these tools to ensure that they enhance the quality of training and, by extension, patient care in a sustainable manner.

### Limitations

This study has the following limitations, which should be considered when interpreting the results. First, it should be noted that the clinical treatment courses in the Department of Orthodontics are practiced and discussed entirely in analogue form, meaning that group A had a significantly better starting point than did group D in terms of approach and handling. However, to that end, a 10-minute practical video introduced the Orthotrainer so that the students could familiarise themselves with the new tool to minimise the familiarity effect of the traditional format.

Furthermore, the monocentric data collection at only one faculty limits the generalisability of the results. The inclusion of students from different semesters also harbours the risk of systematic distortions, for example due to different examination modalities or stress-related differences in performance. We included both seventh-/eighth semester and ninth-/tenth-semester students, potentially differing in orthodontic experience. No stratified analysis was performed; future studies should consider separate analyses or covariate adjustment for year of study. As the analogue and digital assessments were conducted on the same day, without the implementation of a washout period, it is not possible to exclude the possibility of recall bias. It is recommended that subsequent studies consider implementing a longer interval between modalities to mitigate this effect.

Furthermore, the unbalanced gender distribution makes gender-specific analyses more difficult and could have influenced the results. The lack of availability of comparable studies makes it difficult to categorise the results in the existing research context. Finally, the relatively small sample size limits the possibility of making differentiated statements about individual influencing factors.

Although we balanced first- and second-year students across both arms, subgroup sizes remained too small to support robust statistical comparisons by clinical year. Moreover, initial pilot testing revealed some usability challenges with the prototype. While digital evaluation is not a core component of the orthodontic curriculum, external learning influences could still affect usability outcomes, necessitating cautious interpretation [9, 35]. Future studies should examine the impact of individual characteristics, such as stage of clinical training, digital self-efficacy and prior e-learning experience, on diagnostic performance and user satisfaction. This allows for the integration of the app into orthodontic courses as effectively as possible and ensures the achievement of the best possible results in the final orthodontic examination. These factors likely limited peak performance and should guide future software refinements. Despite an overall high SUS rating, students reported specific challenges with the zoom function and mouse navigation, which sometimes hindered precise landmark placement. Suboptimal measurement precision may therefore reflect both user-interface constraints and the learning curve for new controls. Importantly, this study did not include pre-post knowledge testing, follow-up assessments, or a non-intervention control; therefore, no conclusions can be drawn about actual learning gains attributable to Orthotrainer.

## Conclusion

1. The results of this study suggest that e-learning methods for orthodontic case planning can be an effective complement to traditional analogue teaching methods. The web-based platform Orthotrainer has demonstrated equivalent diagnostic accuracy in assessing tooth widths and models, while offering superior reliability in identifying cephalometric landmarks; further studies are needed to confirm these findings under varied conditions and with larger samples.
2. Students rated the platform highly for usability and flexibility, despite minor interface issues, which indicates the potential of digital learning media in dental education. The results suggest that the use of the Orthotrainer enables students to independently develop and practise certain orthodontic content.

While further studies should evaluate individual learning gains and long-term retention, the Orthotrainer’s positive reception and proven advantages indicate strong potential for orthodontic education.

## Supplementary Information

Supplementary Material 1.

## Data Availability

The datasets used and/or analysed during the current study are available from the corresponding author upon reasonable request.
