# Supplementary material for: Orthodontic diagnosis and usability of the web-based e-learning application orthotrainer
Source: BMC Med Educ. 2025 Oct 2;25:1274. doi: 10.1186/s12909-025-07997-9 (PMC12490099; doi:10.1186/s12909-025-07997-9)
Supplement: Supplementary file 1 — Supplementary Material 1. [file 12909_2025_7997_MOESM1_ESM.pdf]

kfo-oc → base 20.01.2025, 14:35

Seite 01

Demographics

D102

1. Alter:

Nur Zahlenwerte eintragen.

D103

2. Geschlecht:

- männlich
- weiblich
- divers

D104

3. Fachsemester:

Nur Zahlenwerte eintragen.

OR01

**4. Unten finden Sie eine weitere Reihe von Aussagen. Bitte geben Sie an, wie weit Sie den
folgenden Aussagen zustimmen bzw. nicht zustimmen.**

	Stimme überhaupt nicht zu	Stimme nicht zu	Stimme weder zu noch lehne ab	Stimme zu	Stimme voll und ganz zu
Ich fühle mich sicher im Umgang mit den Grundfunktionen der Office-Programme	○	○	○	○	○
Ich fühle mich sicher im Umgang mit Software für das Online-Lernen.	○	○	○	○	○
Ich fühle mich sicher in der Nutzung des Internets, um Informationen für das Online-Lernen zu finden oder zu sammeln.	○	○	○	○	○

OR02

5. Unten finden Sie eine weitere Reihe von Aussagen. Bitte geben Sie an, wie weit Sie den folgenden Aussagen zustimmen bzw. nicht zustimmen.

	Stimme überhaupt nicht zu	Stimme nicht zu	Stimme weder zu noch lehne ab	Stimme zu	Stimme voll und ganz zu
Ich führe meinen eigenen Lernplan durch.	○	○	○	○	○
Ich suche Hilfe, wenn ich Lernprobleme habe.	○	○	○	○	○
Ich verwalte meine Zeit gut.	○	○	○	○	○
Ich lege meine Lernziele fest.	○	○	○	○	○
Ich habe höhere Erwartungen an meine Lernleistung.	○	○	○	○	○

OR03

6. Unten finden Sie eine weitere Reihe von Aussagen. Bitte geben Sie an, wie weit Sie den folgenden Aussagen zustimmen bzw. nicht zustimmen.

	Stimme überhaupt nicht zu	Stimme nicht zu	Stimme weder zu noch lehne ab	Stimme zu	Stimme voll und ganz zu
Ich kann meinen eigenen Lernfortschritt steuern.	○	○	○	○	○
Ich werde beim Online-Lernen nicht durch andere Online-Aktivitäten (Sofortnachrichten, Surfen im Internet) abgelenkt.	○	○	○	○	○
Ich habe die Online-Lehrmaterialien entsprechend meinen Bedürfnissen wiederholt.	○	○	○	○	○

OR04

7. Unten finden Sie eine weitere Reihe von Aussagen. Bitte geben Sie an, wie weit Sie den
folgenden Aussagen zustimmen bzw. nicht zustimmen.

	Stimme überhaupt nicht zu	Stimme nicht zu	Stimme weder zu noch lehne ab	Stimme zu	Stimme voll und ganz zu
Ich bin offen für neue Ideen.	○	○	○	○	○
Ich habe die Motivation zu lernen.	○	○	○	○	○
Ich lerne aus meinen Fehlern.	○	○	○	○	○
Ich teile meine Ideen gerne mit anderen.	○	○	○	○	○

OR05

8. Unten finden Sie eine weitere Reihe von Aussagen. Bitte geben Sie an, wie weit Sie den folgenden Aussagen zustimmen bzw. nicht zustimmen.

	Stimme überhaupt nicht zu	Stimme nicht zu	Stimme weder zu noch lehne ab	Stimme zu	Stimme voll und ganz zu
Ich fühle mich sicher im Umgang mit Online-Tools (E-Mail, Social Media), um effektiv mit anderen zu kommunizieren.	○	○	○	○	○
Ich fühle mich sicher, wenn es darum geht, mich in Texten auszudrücken (Gefühle und Humor).	○	○	○	○	○
Ich fühle mich sicher darin, Fragen in Online-Diskussionen zu stellen.	○	○	○	○	○

U101

9. Wie sehr stimmen Sie den folgenden Aussagen zu?

Wählen Sie die passente Antwort

	Stimme vollständig zu				Stimme überhaupt nicht zu
Ich kann mir sehr gut vorstellen, die App regelmäßig zu nutzen.	○	○	○	○	○
Ich empfinde die App als unnötig komplex.	○	○	○	○	○
Ich empfinde die App als einfach zu nutzen.	○	○	○	○	○
Ich denke, dass ich technischen Support brauchen würde, um die App zu nutzen.	○	○	○	○	○
Ich finde, dass die verschiedenen Funktionen der App gut integriert sind.	○	○	○	○	○
Ich finde, dass es in der App zu viele Inkonsistenzen gibt.	○	○	○	○	○
Ich kann mir vorstellen, dass die meisten Leute die App schnell zu beherrschen lernen.	○	○	○	○	○
Ich empfinde die Bedienung als sehr umständlich.	○	○	○	○	○
Ich habe mich bei der Nutzung der App sehr sicher gefühlt.	○	○	○	○	○
Ich musste eine Menge Dinge lernen, bevor ich mit der App arbeiten konnte.	○	○	○	○	○

10. Welche Note geben Sie orthotrainer insgesamt? Z004

○ 1 ○ 2 ○ 3 ○ 4 ○ 5 ○ 6

11. Was hat Ihnen an orthotrainer besonders gut gefallen? Z001

12. Was ließe sich Ihrer Meinung nach an orthotrainer noch verbessern? Z002

13. Sonstige Bemerkungen zu orthotrainer: Z003

Vielen Dank für Ihre Teilnahme!

Wir möchten uns ganz herzlich für Ihre Mithilfe bedanken.

Ihre Antworten wurden gespeichert, Sie können das Browser-Fenster nun schließen.
